# Does home and community-based services use reduce hospital utilization and hospital expenditure among disabled elders? Evidence from China

**DOI:** 10.3389/fpubh.2023.1266949

**Published:** 2023-10-25

**Authors:** Yanling Yi, Junxia Liu, Ling Jiang

**Affiliations:** School of Public Administration, Zhongnan University of Economics and Law, Wuhan, China

**Keywords:** home and community-based services, hospital utilization, hospital expenditure, substitution effect, health effect

## Abstract

**Introduction:**

In the background of aging in place, home and community-based services (HCBS) have been playing an increasingly important role in long-term care (LTC) security systems. However, it is still uncertain whether and how HCBS use affects hospital utilization and the corresponding expenditures.

**Methods:**

Using data from the China Health and Retirement Longitudinal Survey (CHARLS) and the China City Statistical Yearbook, the instrumental variable (IV) approach is applied to identify the causal effects of HCBS use on hospital utilization and hospital expenditure among disabled elders.

**Results:**

We find that HCBS use significantly reduces the probability of being hospitalized, the times of hospitalization, and the length of inpatient stay, as well as the total, out-of-pocket and reimbursement inpatient expenditures, demonstrating not only the substitution impact of HCBS for hospital care but also the effectiveness of medical expenditure control in LTC security systems. Heterogeneity analysis shows that the impacts of HCBS use on hospital utilization and hospital expenditure concentrate on disabled elders who are younger, male, living in urban areas, or from higher-income households; both healthcare and spiritual consolation services have significant negative effects, while the anticipated effects of daily care service use are not supported. The possible mechanisms are the substitution of HCBS for hospital care and the improvements in both the physical and psychological health of disabled elders. However, the mechanism of adverse events decrease is not verified, which needs to be investigated further with more proxy variables.

**Conclusion:**

This study provides empirical evidence that HCBS use can not only reduce hospital utilization and hospital expenditure among disabled elders but also improve their physical and psychological health. Policy designs should emphasize the orientation of HCBS, ensure the fundamental and central position of HCBS in the formal care service system, pay more attention to the accessibility and affordability of HCBS for fragile groups, and diversify and optimize the development of the health service and the spiritual consolation service.

## Introduction

With the ever-increasing aging population, elders with disabilities are growing and their long-term care (LTC) needs have been bringing substantial financial and caregiving burdens to households and governments (1, 2). In OECD countries, LTC expenses were 0.5 to 5 times the median disposable income of older adults aged 65 and over in 2020 (3). Public LTC expenditure was expected to increase from 1.6% of GDP in 2016 to 2.2% in 2040 in the European Union (4). When family caregivers cannot fully meet LTC needs, households will shift to formal care services to supplement and even replace informal care. Home and community-based services (HCBS), as a category of more preferable and less costly services, have been playing an increasingly important role in LTC security systems. On the one hand, HCBS can satisfy the needs of elders better because the majority of them prefer to live in their own homes and have a greater mastery of their daily lives. On the other hand, services supplied in home and community-based settings are much cheaper than in institutions, so HCBS becomes an alternative for governments to control LTC costs more effectively (5). Meanwhile, HCBS can also substitute for hospital care because it can enable hospital inpatients to be discharged sooner (6). A growing body of research has evaluated the impacts of HCBS on hospital utilization and the corresponding expenditures, but no unanimous conclusion has been reached.

Early studies usually apply a comparison-group strategy or a randomized design to evaluate the effects of HCBS (7). However, most find no significant impacts on medical service utilization or expenditures (6). Recently, some studies have supported the effectiveness of HCBS use in reducing hospital utilization. HCBS use is found to be related to a lower probability of hospitalization (8), and the more volume of HCBS use, the less likely it is to be hospitalized, though the effect may fade over time (9). Studies using macro data at the district level also provide encouraging evidence. Forder (10) reported that every additional £1 expended on care home services can reduce hospital costs by approximately £0.35 and vice versa, implying that care home services and hospital services can partially substitute for each other. Gaughan et al. (11) found that an increase in care home beds can reduce delayed discharges significantly. Still, there are doubts about the negative effects of HCBS use on hospital utilization. Hermiz et al. (12) found that although home visits by community nurses and preventive care by general practitioners improve the health knowledge and life quality of patients discharged after acute care, these services have no effect on readmission to the hospital or visits to general practitioners. Even more, Deraas et al. (13) reported that LTC rates (total number of LTC recipients per 1,000 inhabitants) have a weak positive adjusted relation to HD rates (hospital days per 1,000 inhabitants). These inconsistent conclusions may arise from various types of HCBS, diverse target populations, different research designs, etc., making the effects of HCBS on hospital utilization and expenditure unclear, and many of the studies are also limited to small sample sizes and lack of concern for endogeneity. The two studies of Forder (10) and Gaughan et al. (11) selected the instrumental variable (IV) approach to address endogeneity, but both use macro data at the district level, not representative individual data. Therefore, combining nationally representative individual data from the China Health and Retirement Longitudinal Survey (CHARLS) and city-level macro data from the China City Statistical Yearbook, this study investigates the impacts of HCBS use on hospital utilization and expenditure among disabled elders.

HCBS use can affect hospital utilization directly and indirectly. First, as a substitute for hospital care, HCBS can be used to replace hospital care directly. If HCBS is available and cheaper, patients will transfer from hospitals to homes or community centers when ready to be discharged medically, which will attenuate hospital bed-blocking. Second, HCBS use can indirectly reduce hospital admissions and readmissions by better meeting LTC needs and reducing adverse events. Disabled elders are more likely to be hospitalized with unmet needs, but this situation disappears 6 weeks after enrolling All-inclusive Care program (14). Older patients are vulnerable (15), of whom 20% experience adverse events during the early several weeks after discharge (16), which can lead to readmissions. Adequate care can be very helpful for them to manage well (17). Third, through its effects on health, HCBS use can indirectly influence hospital utilization. The supply and utilization of HCBS can increase the physical and psychological health of the older adults (18), which can decrease hospital utilization.

The IV approach is applied to identify the causal effects of HCBS use on hospital utilization and hospital expenditure. We first demonstrate that HCBS use significantly reduces the probability of being hospitalized, the times of hospitalization and length of inpatient stay, and the total, out-of-pocket (OOP), and reimbursement inpatient expenditures. Then, we compare heterogeneous effects of HCBS use across individual characteristics and service categories and find that the effects concentrate on the disabled elders who are younger, male, living in urban areas, or from higher-income households, and both health services and spiritual consolation services have significant negative impacts on hospital utilization and hospital expenditure, while the anticipated effects of daily care service use are not supported. Finally, we demonstrate that the HCBS use by disabled elders is beneficial to their physical and psychological health, which can further reduce their hospital utilization and hospital expenditure.

This study makes several contributions to the literature as follows. First, using nationally representative individual data from the recent wave of CHARLS, this study provides empirical evidence from the individual level to support the substitution of LTC services for medical care and the medical cost control effect of HCBS. Compared to macro data, there is abundant information on demographic and socioeconomic factors, which are controlled for when they are potential confounders, helping us to estimate the effects more precisely. Second, by applying the IV method and controlling for city-level confounders from the China City Statistical Yearbook, this study addresses the potential endogeneity of HCBS use more carefully. Third, in addition to previous studies, this study investigates the heterogeneous effects of HCBS use on different groups with individual characteristics and the heterogeneous impacts of three different types of HCBS use, which provide empirical evidence for the further optimization of service items. Fourth, this study argues that the possible mechanisms are the substitution of HCBS for hospital care and the improvement of both the physical and psychological health of disabled elders.

## Materials and methods

### Data source

Individual data used in this study are from the CHARLS, while city-level data are from the China City Statistical Yearbook. CHARLS is an interdisciplinary longitudinal survey conducted by the National School of Development at Peking University, and CHARLS has been collecting abundant information from a nationally representative sample of Chinese residents aged 45 years and above on their demographic characteristics, family structure, physical and psychological health, medical services utilization and expenditures, household income, and consumption. The baseline survey was conducted in 2011, and three follow-up surveys were implemented in 2013, 2015, and 2018, with a final sample of 19,000 respondents from 12,400 households. We only use the recent wave of data in 2018 because only in this wave there was information on HCBS use, the key independent variable in this study. The China City Statistical Yearbook contains primary statistical data on the social and economic development of more than 650 cities in China, and we use city-level information in 2018 on population, economy, and public health conditions.

### Study sample

The study sample consists of disabled elders who are 60 years or older. Respondents were inquired about their difficulties in ADLs, including dressing, bathing, eating, getting into or out of bed, using the toilet, and controlling urination, and in instrumental ADLs, such as doing household chores, preparing hot meals, shopping for groceries, making phone calls, taking medications, and managing money. Individuals who reported having difficulties in any of those activities are defined as disabled in this study. We first identify 6,734 disabled people and then drop those under the age of 60 years and those with missing information on independent variables. Finally, 4,544 valid samples remain.

### Variables

Outcome variables in this study are selected from two aspects: hospital utilization and hospital expenditure. Following previous studies (19–21), we measure hospital utilization by three variables: hospital admission, hospitalizations, and length of inpatient stay, and select multiple variables of hospital expenditure: total inpatient expenditure, OOP inpatient expenditure, and reimbursement inpatient expenditure. Hospital admission is assessed with the question “Have you received inpatient care in the past year?” and takes the value 1 if the answer is “yes” and the value 0 if the answer is “no.” The variable hospitalizations is a count variable that measures the number of times a disabled older adult had been hospitalized during the past year. Length of inpatient stay is also a count variable representing the number of nights a disabled older adult had spent for the last hospitalization in the past year. For variables of hospital expenditure, the respondents were required to report the total medical cost for all the inpatient care they had received during the past year and the OOP part of it, so the reimbursement inpatient expenditure is the difference between the two.

The key independent variable is HCBS use, constructed according to the question, “Have you ever received the following home and community care services?” The answers include daycare centers, nursing homes, senior dining tables, regular physical examinations, onsite visits, family beds, community nursing, health management, entertainment, and the option of “other.” HCBS use is coded as 1 if a respondent reported having used any of the above services and 0 if the respondent had not.

Covariates are chosen from two aspects: individual level and city level, to account for possible confounding factors. While demographic and socioeconomic variables at the individual level include age, female, marital status (married and living with one's spouse = 1, other = 0), education level (primary school and below = 0, junior high school and above = 1), residence (urban = 1, rural = 0), number of living children, UEBMI (having urban employee basic medical insurance = 1, otherwise = 0), URRBMI (having urban and rural residents basic medical insurance = 1, otherwise = 0), and household income per capita, city-level covariates contain natural growth rate of population, GDP per capita, fiscal expenditure per capita, number of hospital beds per 1,000 inhabitants, and number of doctors per 1,000 inhabitants.

### The ordinary least squares model

We use the ordinary least squares (OLS) model to estimate the association between HCBS use and hospital utilization. The equation is as follows:


(1) $$\begin{array}{l} Y_{i}=\alpha_{0}+\alpha_{1}HCBS_{i}+\alpha_{2}X_{i}+\alpha_{3}W_{c}+u_{i} \end{array}$$

*Y*_*i*_ represents the potential outcomes of hospital utilization or hospital expenditure for the disabled elder *i*, including six indicators: hospital admission, hospitalizations, length of inpatient stay, total inpatient expenditure, OOP inpatient expenditure, and reimbursement inpatient expenditure. *HCBS*_*i*_ is a dummy variable indicating whether the disabled elder *i* had used HCBS in the past year. While *X*_*i*_ is a set of individual covariates including age, age's square, female, marital status, education level, urban residence, number of living children, UEBMI, URRBMI, and household income per capita, *W*_*c*_ contains city-level variables such as natural growth rate of population, GDP per capita, fiscal expenditure per capita, number of hospital beds per 1,000 inhabitants, and number of doctors per 1,000 inhabitants.

### Instrumental variable approach

The endogeneity of HCBS use should be considered when estimating the impacts of HCBS use on hospital utilization and hospital expenditure. The endogeneity may stem from the two following sources: first, there may be bias from omitted variables. Factors such as the quality of LTC, the quality of medical care services, individual preference, and so on may simultaneously affect the decision between HCBS and hospital care, which are unobservable or unavailable. Second, there may be bias from self-selection. Whether a disabled elder used HCBS is decided by oneself or one's family, which may lead to systematic differences between the sample who had used HCBS and the one who had not. In the following section of Descriptive Statistics, we find significant differences in age, marital status, educational level, and GDP per capita between the used and unused samples, indicating that there may be endogeneity caused by self-selection.

We apply the IV approach to address the potential endogeneity of HCBS use. Following previous studies (22–24), we use *Rate*_*city*_, which represents the average utilization rate of HCBS in the city where the disabled elder *i* lived, as the IV of HCBS use. First, *Rate*_*city*_ is strongly related to the HCBS use by a disabled elder. Traditionally, most of the disabled elders in China depend on informal care, especially the care provided by spouses and children, to meet their LTC needs, and both the supply and utilization of HCBS are limited. The increase in HCBS use in recent years is mainly driven by the pilot reform of community and home-based care services for older adults, which is declared to start officially in 2016, aiming to build a multi-level care service system that is based on home care, supported by community care, and supplemented by institution care. The HCBS use by a disabled elder is affected by the supply of those services in the city and the demonstration effect of other people; therefore, *Rate*_*city*_ is closely associated with one's HCBS use. Second, *Rate*_*city*_ does not directly influence the hospital utilization and hospital expenditure of a disabled elder. As discussed just now, the variation in *Rate*_*city*_ among various cities is mainly affected by exogenous institutional reform, making *Rate*_*city*_ independent of other factors that influence the hospital utilization and hospital expenditure of a disabled elder. Moreover, by controlling for city-level covariables and carefully excluding the individual self when calculating *Rate*_*city*_, the independence of *Rate*_*city*_ is further guaranteed.

Specifically, we select two-stage least squares (2SLS) for IV estimation. The equations are as follows:


(2) $$\begin{array}{lll} HCBS_{i} & = & \beta_{0}+\beta_{1}Rate_{city}+\beta_{2}X_{i}+\beta_{3}W_{c}+v_{i} \end{array}$$

(3) $$\begin{array}{lll} Y_{i} & = & \gamma_{0}+\gamma_{1}\hat{HCBS_{i}}+\gamma_{2}X_{i}+\gamma_{3}W_{c}+\varepsilon_{i} \end{array}$$

While equation (2) is the first stage regression of 2SLS, equation (3) represents the second stage. Our primary interest is γ_1_, which estimates the causal effects of HCBS use on hospital utilization or hospital expenditure.

## Results

### Descriptive statistics

Table 1 shows descriptive statistics for the full sample, the used subsample, and the unused subsample. For the dependent variables, 27% of the disabled elders reported having been hospitalized during the past year, and the average hospitalizations and length of inpatient stay in the past year are 0.445 and 3.033, while the means of total, OOP, and reimbursement inpatient expenditures are RMB 3,618, 1,724, and 1,714 yuan, respectively. Except for hospital admission, the average hospitalizations, length of inpatient stay, and each inpatient expenditure of the used sample are slightly lower than those of the unused subsample. However, there are no significant differences in all the outcome variables between the two subsamples, according to the results of the *t*-test.

**Table 1:** Descriptive statistics.

**Variables**	**Full**			**Used**	**Unused**
	**Observations**	**Mean/%**	**Standard deviation**	**Mean/%**	**Mean/%**
**Dependent variables**					
Hospital admission	4,544	0.270	0.444	0.270	0.270
Hospitalizations	4,544	0.445	0.877	0.440	0.446
Length of inpatient stay	4,544	3.033	6.264	2.778	3.089
Total inpatient expenditure	4,443	3,648	10,615	3,365	3,710
OOP inpatient expenditure	4,443	1,724	5,276	1,632	1,743
Reimbursement inpatient expenditure	4,443	1,714	5,276	1,577	1,743
**The key independent variable and IVs**					
HCBS	4,544	0.181	0.385	1.000	0.000
*Rate*_city_	4,544	0.180	0.129	0.253^***^	0.164
*Rate*_comty_	4,532	0.179	0.170	0.270^***^	0.160
**Covariates**					
Age	4,544	71.39	7.641	72.54^***^	71.14
Female	4,544	0.613	0.487	0.618	0.612
Marital status	4,544	0.713	0.452	0.688^*^	0.719
Education level	4,544	0.127	0.333	0.107^*^	0.132
Urban residence	4,544	0.155	0.362	0.146	0.157
Number of living children	4,544	3.404	1.497	3.458	3.392
UEBMI	4,544	0.094	0.291	0.096	0.093
URRBMI	4,544	0.862	0.345	0.872	0.859
Household income per capita	4,544	9,617	13,125	9,945	9,545
GDP per capita	4,544	52,515	30,575	54,965^**^	51,975
Fiscal expenditure per capita	4,544	9,982	5,788	10,177	9,938
Number of hospital beds per 1,000 inhabitants	4,544	4.431	1.636	4.488	4.419
Number of doctors per 1,000 inhabitants	4,544	2.373	0.993	2.405	2.366
Natural growth rate of population	4,544	6.345	4.175	6.443	6.323

The t-test is applied for pairwise comparisons between the HCBS used sample and unused sample respectively.

***, **, and * mean the significance levels of 1%, 5%, and 10%, respectively.

For the key independent variables and IVs, 18.1% of the disabled elders reported having used HCBS in the past year, and *Rate*_*city*_ and *Rate*_*comty*_ are 18.0% and 17.9%, respectively. The *Rate*_*city*_ and *Rate*_*comty*_ of the used sample are much higher than those of the unused sample, i.e., 9 and 11.1 percentage points higher, respectively, indicating that whether a disabled elder chooses to use HCBS is strongly related to the HCBS use by other people in the same community and the development of HCBS reform in the city.

For covariates, the disabled elders have an average age of 71.39, of which 61.3% are women, 71.3% are married and living with their spouse, and 15.5% live in urban areas. The average means or proportions of the used subsample on most of the variables are slightly higher than the unused subsample except for three, namely marital status, education level, and urban residence, while the results of the *t*-test show that there are significant differences between the two subsamples in age, marital status, education level, and GDP per capita, suggesting that the used subsample is much different from the unused subsample and there is endogeneity from self-selection.

### OLS estimates

Table 2 represents the OLS estimates of the effects of HCBS use on hospital utilization and hospital expenditure. Although HCBS use is negatively associated with all the outcome variables of hospital utilization and hospital expenditure, the estimated coefficients of HCBS use are not statistically significant except for one, namely the coefficient of HCBS use on length of inpatient stay. In particular, HCBS use by disabled elders is related to a drop of 0.41 nights in the inpatient stay, and the coefficient is significant at the confidence level of 10%. Overall, the results of OLS show that HCBS use can significantly reduce the length of inpatient stays for disabled elders but has no impact on the other two variables of hospital utilization or all three outcomes of hospital expenditure. However, the OLS estimates may be biased because HCBS use might be endogenous according to the previous analysis, and further tests are required.

**Table 2:** OLS estimates of the effects of HCBS use on hospital utilization and hospital expenditure.

**Variables**	**(1)**	**(2)**	**(3)**	**(4)**	**(5)**	**(6)**
	**Hospital admission**	**Hospitalizations**	**Length of inpatient stay**	**Total inpatient expenditure**	**OOP inpatient expenditure**	**Rei. inpatient expenditure**
HCBS	−0.0092	−0.0196	−0.413^*^	−0.152	−0.110	−0.0364
	(0.0171)	(0.0330)	(0.224)	(0.152)	(0.137)	(0.136)
Individual covariates	Yes	Yes	Yes	Yes	Yes	Yes
City-level covariates	Yes	Yes	Yes	Yes	Yes	Yes
Observations	4,544	4,544	4,544	4,443	4,443	4,443
R-squared	0.028	0.025	0.024	0.029	0.025	0.033

Robust standard errors are reported in parentheses.

^***^, ^**^, and

* mean the significance levels of 1%, 5%, and 10%, respectively. Individual covariates include age, age's square, female, marital status, education level, urban residence, number of living children, UEBMI, URRBMI and household income per capita. City-level covariates contain natural growth rate of population, GDP per capita, fiscal expenditure per capita, number of hospital beds per 1,000 inhabitants, and number of doctors per 1,000 inhabitants.

### IV estimates

Table 3 shows the estimates of IV regressions. We first pay attention to the results of validity tests on IV. The *P*-values of the Durbin-Wu-Hausman test range from 0.023 to 0.078, rejecting the assumption that HCBS use is an exogenous variable in all IV regressions at the confidence levels of 5% or 10%. In the first-stage regressions, the coefficients of HCBS are all positive at the confidence level of 1%, illustrating that our IV *Rate*_*city*_ is strongly associated with the endogenous key independent variable of HCBS use. Besides, the F-statistics in all the first-stage regressions are far >10, also rejecting the assumption of weak IV. Therefore, the selection of *Rate*_*city*_ as IV is necessary and appropriate.

**Table 3:** IV estimates of the effects of HCBS use on hospital utilization and hospital expenditure.

**Variables**	**(1)**	**(2)**	**(3)**	**(4)**	**(5)**	**(6)**
	**Hospital admission**	**Hospitalizations**	**Length of inpatient stay**	**Total inpatient expenditure**	**OOP inpatient expenditure**	**Rei. inpatient expenditure**
**Panel A: the second stage**						
HCBS	−0.144^**^	−0.243^**^	−2.146^**^	−1.475^***^	−1.239^**^	−1.141^**^
	(0.063)	(0.122)	(0.853)	(0.567)	(0.511)	(0.505)
Individual covariates	Yes	Yes	Yes	Yes	Yes	Yes
City-level covariates	Yes	Yes	Yes	Yes	Yes	Yes
**Panel B: the first stage**						
*Rate*_city_	0.768^***^	0.768^***^	0.768^***^	0.763^***^	0.763^***^	0.763^***^
	(0.0516)	(0.0516)	(0.0516)	(0.0522)	(0.0522)	(0.0522)
Individual covariates	Yes	Yes	Yes	Yes	Yes	Yes
City-level covariates	Yes	Yes	Yes	Yes	Yes	Yes
First-stage F-statistics (*P*-value)	221.43 (0.000)	221.43 (0.000)	221.43 (0.000)	212.98 (0.000)	212.98 (0.000)	212.98 (0.000)
Durbin-Wu-Hausman (*P*-value)	4.431 (0.035)	3.101 (0.078)	3.666 (0.056)	5.205 (0.023)	4.624 (0.032)	4.587 (0.032)
Observations	4,544	4,544	4,544	4,443	4,443	4,443
R-squared	0.014	0.015	0.013	0.013	0.010	0.019

Notes are the same as specified in Table 2.

In particular, the results of the second stage of IV regressions show that for hospital utilization, HCBS use is associated with a decrease of 14.4 percentage points in the proportion of hospital admissions, a reduction of 0.243 times in hospitalizations, and a drop of 2.146 days in the length of inpatient stay, implying that HCBS use by disabled elders significantly reduces their hospital utilization and that HCBS are substitutes for hospital services; for hospital expenditure, HCBS use reduces total, OOP, and reimbursement inpatient expenditures by 148%, 124%, and 114%, respectively. In summary, HCBS use can not only reduce hospital utilization of disabled elders and relieve hospital bed-blocking but also can be very helpful in controlling medical expenses effectively and reducing the burden of medical insurance funds.

Comparing the results in Tables 2, 3, we can see that while five in six estimates of OLS regressions are insignificant and one is significant at the confidence level of 10%, all IV estimates become statistically significant at the confidence level of 5% or 1%. Furthermore, all the coefficients of HCBS use of IV regressions are far greater than those of OLS regressions, suggesting that ignoring the endogeneity of HCBS use will lead to an underestimation.

### Robustness

Following the strategy of Feng (24), we further test the robustness of our baseline IV estimates and the exogeneity of IVs by adding another IV *Rate*_*comty*_ and changing 2SLS to GMM (generalized method of moments), of which the results are revealed in Table 4. The results show that HCBS use is related to a decrease of 12.8 percentage points in the proportion of hospital admissions, a reduction of 0.203 in hospitalizations, and a drop of 1.956 days in the length of inpatient stay. HCBS use also significantly reduces total, OOP, and reimbursement expenditures by 132.7%, 113.8%, and 100.7%, respectively. With the added IV *Rate*_*comty*_, the results are very similar to those with one IV, both in significance and magnitude, implying that our baseline IV estimates are reliable. Besides, the statistics of Hansen J range from 0.294 to 2.300 and are all not significant, suggesting that both the two IVs are exogenous variables.

**Table 4:** Robustness: estimates with two IVs.

**Variables**	**(1)**	**(2)**	**(3)**	**(4)**	**(5)**	**(6)**
	**Hospital admission**	**Hospitalizations**	**Length of inpatient stay**	**Total inpatient expenditure**	**OOP inpatient expenditure**	**Rei. inpatient expenditure**
**Panel A: the second stage**						
HCBS	−0.128^**^	−0.203^*^	−1.956^**^	−1.327^**^	−1.138^**^	−1.007^**^
	(0.0599)	(0.118)	(0.827)	(0.545)	(0.492)	(0.487)
Individual covariates	Yes	Yes	Yes	Yes	Yes	Yes
City-level covariates	Yes	Yes	Yes	Yes	Yes	Yes
**Panel B: the first stage**						
*Rate*_city_	0.552^***^	0.552^***^	0.552^***^	0.560^***^	0.560^***^	0.560^***^
	(0.077)	(0.077)	(0.077)	(0.077)	(0.077)	(0.077)
*Rate*_comty_	0.232^***^	0.232^***^	0.232^***^	0.218^***^	0.218^***^	0.218^***^
	(0.057)	(0.057)	(0.057)	(0.057)	(0.057)	(0.057)
Individual covariates	Yes	Yes	Yes	Yes	Yes	Yes
City-level covariates	Yes	Yes	Yes	Yes	Yes	Yes
First-stage F-statistic (*P*-value)	125.01 (0.000)	125.01 (0.000)	125.01 (0.000)	119.35 (0.000)	119.35 (0.000)	119.35 (0.000)
Durbin-Wu-Hausman (*P*-value)	3.728 (0.054)	1.902 (0.168)	2.960 (0.085)	4.404 (0.036)	4.141 (0.042)	3.760 (0.053)
Hansen J statistic (*P*-value)	0.482 (0.487)	2.300 (0.129)	0.720 (0.396)	0.656 (0.418)	0.294 (0.588)	0.791 (0.374)
Observations	4,532	4,532	4,532	4,432	4,432	4,432
R-squared	0.017	0.019	0.015	0.016	0.013	0.022

Notes are the same as specified in Table 2.

### Heterogeneity

**Figure 1:**
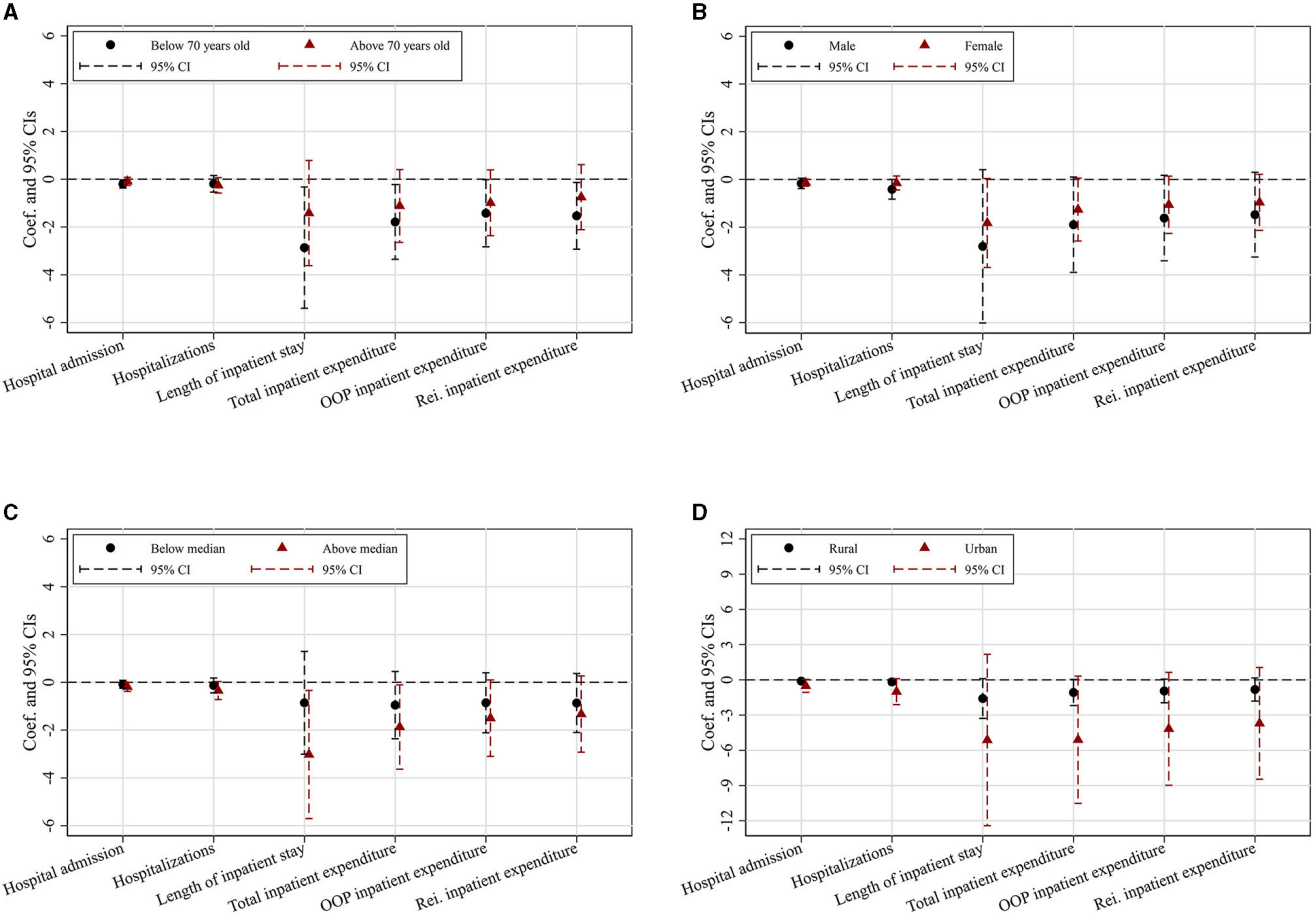
Heterogeneous effects of HCBS use across individual characteristics. The Y-axis represents the estimated coefficients of HCBS use and their respective confidence intervals, while the X-axis indicates six outcomes of hospital utilization and hospital expenditure. Each regression controls for individual and city-level covariates as specified in Table 2. **(A)** By age. **(B)** By gender. **(C)** By household income per capita. **(D)** By urban/rural residence.

The effects of HCBS use on hospital utilization and hospital expenditure may differ across different individual characteristics, and different types of HCBS may also have heterogeneous performances. Therefore, again using *Rate*_*city*_ as IV, we estimate the heterogeneous effects of HCBS use. Figure 1 shows the heterogeneous effects of HCBS use on different groups of disabled elders, divided by some variables of individual characteristics, while Table 5 represents the heterogeneous effects of three different types of HCBS use on hospital utilization and hospital expenditure.

**Table 5:** The heterogeneous effects of three different types of HCBS use.

**Variables**	**(1)**	**(2)**	**(3)**	**(4)**	**(5)**	**(6)**
	**Hospital admission**	**Hospitalizations**	**Length of inpatient stay**	**Total inpatient expenditure**	**OOP inpatient expenditure**	**Rei. inpatient expenditure**
**Panel A**						
Daily care service use	−0.489	1.669	2.640	−4.114	−2.968	−3.096
	(1.052)	(2.218)	(14.61)	(9.358)	(8.812)	(8.290)
First-stage *F*-statistics	2.27	2.27	2.27	2.06	2.06	2.06
(*P*-value)	(0.104)	(0.104)	(0.104)	(0.128)	(0.128)	(0.128)
Observations	4,532	4,532	4,532	4,432	4,432	4,432
R-squared	0.021	0.003	0.022	0.026	0.023	0.031
**Panel B**						
Health service use	−0.146^**^	−0.246^**^	−2.175^**^	−1.483^***^	−1.246^**^	−1.147^**^
	(0.0635)	(0.124)	(0.864)	(0.570)	(0.514)	(0.508)
First-stage *F*-statistics	219.09	219.09	219.09	213.52	213.52	213.52
(*P*-value)	(0.000)	(0.000)	(0.000)	(0.000)	(0.000)	(0.000)
Observations	4,544	4,544	4,544	4,443	4,443	4,443
R-squared	0.016	0.016	0.015	0.013	0.011	0.019
**Panel C**						
Spiritual comfort service use	−0.847^**^	−1.427^*^	−12.62^**^	−8.987^**^	−7.554^**^	−6.955^**^
	(0.399)	(0.761)	(5.546)	(3.839)	(3.423)	(3.340)
First-stage *F*-statistics	26.04	26.04	26.04	23.74	23.74	23.74
(*P*-value)	(0.000)	(0.000)	(0.000)	(0.000)	(0.000)	(0.000)
Observations	4,544	4,544	4,544	4,443	4,443	4,443
R-squared	−0.023	−0.009	−0.039	−0.036	−0.032	−0.016

Robust standard errors are reported in parentheses. ^***^, ^**^, and ^*^ mean the significance levels of 1%, 5%, and 10%, respectively. Each regression controls for individual and city-level covariates as specified in Table 2.

Figure 1A shows the heterogeneous effects of HCBS use on hospital utilization and hospital expenditure between younger and older disabled elders. We classify disabled elders under 70 years old into the younger group, making those aged 70 years and above fall into the older group. For the younger group, HCBS use has significant negative impacts on all the outcome variables of hospital utilization and hospital expenditure except hospitalizations, while for the older group, there are no significant expected effects. The effects of HCBS use on hospital utilization and hospital expenditure concentrate on the younger disabled elders.

Figure 1B demonstrates that for both female and male disabled elders, HCBS use can reduce their hospital utilization and hospital expenditure. However, while five in six coefficients of HCBS use for the male sample are significant, only two for the female sample. Furthermore, all the coefficients of HCBS use for men appear to be greater than those for women, indicating that the effects of HCBS use on hospital utilization and hospital expenditure are stronger among male disabled elders.

Figure 1C represents the heterogeneous effects between the lower- and higher-income groups. The full sample is divided by household income per capita. For the higher-income group (above the 50th percentile), the impacts of HCBS on various outcome variables are all significant, but not for the lower-income group (less than or equal to the 50th percentile). Furthermore, the estimates for the higher-income group seem to be larger than those for the lower-income group, implying that HCBS use is more effective for the higher-income group in reducing hospital utilization and controlling hospital expenditure.

Figure 1D shows the heterogeneous effects across urban and rural residences. Disabled elders in urban and rural areas have reduced hospital utilization and hospital expenditure because of HCBS use. Specifically, HCBS use significantly reduces the probability of hospital admission, hospitalizations, total inpatient expenditure, and OOP inpatient expenditure among urban disabled elders, while among rural ones, HCBS use has significant negative effects on the length of inpatient stay, total inpatient expenditure, and OOP inpatient expenditure. However, all the coefficients of HCBS use for the urban group seem to be greater than those for the rural group, whether significant or not, implying that the effects of HCBS use on hospital utilization and hospital expenditure are stronger for the urban disabled elders.

Panels A–C in Table 5 represent, respectively, the effects of the use of three different types of HCBS, namely daily care service use, health service use, and spiritual consolation service use, on hospital utilization and hospital expenditures. The questionnaire of CHARLS enquired the respondents about the HCBS they had received in the last year, and the variable daily care service use is coded as 1 if respondents reported that they had used any service of daycare centers, nursing homes, senior dining tables, etc.; the variable health service use takes the value 1 if they reported using any of regular physical examination, onsite visits, community nursing, and health management; and the variable spiritual consolation service use is equal to 1 if they reported participating in community entertainments. For those who did not report using the specific kind of HCBS, the corresponding variable takes the value 0.

The results in Table 5 show that both health service use and spiritual consolation service use have significant negative effects on all six outcomes of hospital utilization and hospital expenditure, and all the estimated coefficients of spiritual consolation service use are greater than those of health service use, implying that mental health is of particular importance to the disabled elders. However, no significant evidence supports the anticipated impacts of daily care service use. The IV *Rate*_*city*_, which is always significantly related to the endogenous key independent variable in other IV regressions, is a weak IV here, and this problem remains even when we use the two IVs and change 2SLS to LIML (limited-information maximum likelihood), as shown in panel A. There may be two reasons. First, daily care services require a relatively lower level of technical ability, so they are not substitutes for hospital inpatient care with high-level professional knowledge and skills. Second, very few respondents reported using daily care services, i.e., only 0.5% of the full sample, resulting in too few variations in the key independent variable to influence the dependent variables. Whatever the causes are, they need to be examined further.

### Mechanisms

As discussed in the “Introduction” section, the HCBS use by disabled elders can influence hospital utilization and hospital expenditure, both directly and indirectly. Without information on LTC care delayed discharges, we cannot directly test the direct substitution effect. However, consistent with the study of Wang and Feng (27), the results of age and gender heterogeneity provide evidence for the direct substitution impact because the effects are stronger among younger and male disabled elders, who have average lower disability levels. Limited by the availability of data, we only test the impacts of HCBS use on self-reported health, depression, and fall here, and the results are revealed in Table 6. The results show that HCBS use is related to a reduction of 13.7 percentage points in self-reporting fair/poor health and a drop of 22.1 percentage points in depression, indicating that HCBS use improves both the physical and psychological health of disabled elders, but there is no evidence to support the expected effects on fall, so the mechanism of adverse event decrease is not illustrated, which needs to be investigated further with more proxy variables.

**Table 6:** Mechanisms.

**Variables**	**(1)**	**(2)**	**(3)**
	**Health**	**Depression**	**Fall**
HCBS	−0.137^**^	−0.221^***^	−0.000276
	(0.0611)	(0.0782)	(0.0674)
First-stage *F*-statistics (*P*-value)	198.07	197.70	220.69
	(0.000)	(0.000)	(0.000)
Observations	3,925	3,804	4,540
R-squared	−0.009	0.018	0.012

Notes are the same as specified in Table 5.

## Discussion

Using data from the 2018 wave of CHARLS and the China City Statistical Yearbook published in 2019, this study examines the causal effects of HCBS use on hospital utilization and hospital expenditure by IV approach.

We demonstrate that the HCBS use by disabled elders in China reduces both their hospital utilization and hospital expenditure, indicating that HCBS are substitutes for hospital inpatient care and that HCBS use can be very helpful in alleviating the care burden of hospitals and controlling the ever-increasing medical expenses. Our findings are consistent with previous studies assessing the negative impacts of formal LTC on hospital utilization and hospital expenditure (10, 11), which use macro data at the district level. However, some other studies find less effect of community-based health services on medical services (6, 12) and even a positive relationship between the two (13). This study adds new evidence from the individual level to the literature to support the significant negative effects of LTC on reducing the utilization of medical services and the corresponding expenses.

We find that the impacts of HCBS use on hospital utilization and hospital expenditure concentrate on disabled elders who are younger or male. Previous studies illustrate that the disability degree of the elders will increase with age (25–28), women have poorer health than men (29, 30), and the disability status is more severe among women elders than men (31). The higher the level of disability an elder has, the more professional medical care one needs. For the lower-level disabled elders, their requirements for high-level professional care services are much fewer, which can be met better by HCBS. On the contrary, the higher-level disabled elders may need more high-level professional medical care services, which usually cannot be supplied well by community centers. Besides, we also found more pronounced impacts among disabled elders from higher-income households or living in urban areas, which may be related to their higher capacity to pay, the relatively fewer medical and formal care resources in rural areas, and even the stronger concept of relying on family members, especially children, for LTC care of rural elders. These findings call our attention to fragile groups. More precise measures should be developed to help them access and afford HCBS, enabling them to live in their own homes longer.

Furthermore, there are kind-specific heterogeneous effects of HCBS use on hospital utilization and hospital expenditure. Specifically, the significant influences of spiritual consolation service use seem to be greater than those of health service use, while there is no significant evidence to support the anticipated effects of daily care service use. These findings provide evidence to emphasize the psychological well-being of disabled elders and the necessity of diversifying and optimizing various service items of HCBS to satisfy the various needs of disabled elders, especially their spiritual needs.

The possible mechanisms are the direct substitution of HCBS on hospital care and health improvement, which are in line with that of Wang and Feng (25), who argued that the substitution of LTCI on inpatient care utilization and impatient expenditure concentrate more on elders with lower disability levels, and that of Lv and Zhang (18), who illustrated that HCBS use is beneficial to the health of old adults. However, we find no evidence to support the mechanism of adverse events decreasing, which is not consistent with previous studies by Sands et al. (14) and Bragstad et al. (17). It is necessary to investigate further with more proxy variables as there is only one in our study.

This study has several limitations. First, we use hospital admission, hospitalizations, and length of inpatient stay instead of hospital readmissions, avoidable hospital utilization, or social care delayed discharges because there is no information on these variables in our data. Second, it should be interpreted with caution that the estimated effects of HCBS use on the length of inpatient stay for the variable here refer to only the nights of the last time spent in the hospital rather than the past year a respondent had spent in the hospital. Third, there may still be violations of the assumptions of the IV method even if we have illustrated the exogeneity of our IV *Rate*_*city*_ conceptually and empirically. Moreover, there is a problem of weak IV when we estimate the effects of daily care service use on hospital utilization and hospital expenditure, which calls for a more comprehensive investigation in the future.

## Conclusion

This study provides empirical evidence that HCBS use can not only reduce hospital utilization and hospital expenditure of disabled elders but also improve their physical and psychological health, implying that HCBS can help achieve the healthcare goal of ensuring healthy lives for all by 2030, which was adopted as the United Nations Post-2015 Sustainable Development Goals (32), with fewer costs. Furthermore, HCBS use can satisfy the needs of elders much better by maintaining a virtuous cycle, i.e., HCBS use, which can promote the health status of disabled elders, enables them to live in their own homes and maintain some control of their daily lives, and with improved health status, disabled elders can participate more in the household's decision-making activities (33) and therefore maintain more control of their daily lives and live in their own home much longer. Policy designs should emphasize the orientation of HCBS and ensure the fundamental and central position of HCBS in the formal care service system. In addition, more attention should be paid to the accessibility and affordability of HCBS for fragile groups and the diversification and optimization of the development of various service items, especially the health service and the spiritual consolation service, in order to fully utilize its role in improving the wellbeing of elders and controlling medical expenses.

## Data availability statement

Publicly available datasets were analyzed in this study. This data can be found here: https://charls.pku.edu.cn/en.

## Ethics statement

Ethical approval for all the CHARLS waves was granted from the Institutional Review Board at Peking University. The IRB approval numbers are IRB00001052-11015 and IRB00001052-11014. The studies were conducted in accordance with the local legislation and institutional requirements. Written informed consent for participation was not required from the participants or the participants' legal guardians/next of kin because as the datasets of CHARLS are publicly available, ethical approval was not needed for this study.

## Author contributions

YY: Data curation, Conceptualization, Formal analysis, Writing—original draft. JL: Supervision, Writing—review and editing. LJ: Data curation, Writing—review and editing.

## References

[1] Guets W Behera DK. Does disability increase households' health financial risk: evidence from the Uganda demographic and health survey. Global Health Res Policy. (2022) 7:1–8. 10.1186/s41256-021-00235-x 34983699 PMC8728967

[2] Arai Y Zarit SH. Exploring strategies to alleviate caregiver burden: effects of the national long-term care insurance scheme in Japan. Psychogeriatrics. (2011) 11:183–9. 10.1111/j.1479-8301.2011.00367.x 21951960

[3] Liu H Ma J Zhao L. Public long-term care insurance and consumption of elderly households: evidence from China. J Health Econ. (2023) 90:102759. 10.1016/j.jhealeco.2023.102759 37146408

[4] Barber SL van Gool K Wise S Wood M Or Z Penneau A. Pricing Long-Term Care for Older Persons. Geneva: World Health Organization. (2021).

[5] Shirk C. Rebalancing Long-term Care: The Role of the Medicaid HCBS Waiver Program. (2006).

[6] Wooldridge J Schore J. The evaluation of the National Long Term Care Demonstration. The effect of channeling on the use of nursing homes, hospitals, and other medical services. Health Serv Res. (1988) 23:119. 3130323 PMC1065492

[7] Carcagno GJ Kemper P. The evaluation of the National Long Term Care Demonstration. 1 An overview of the channeling demonstration and its evaluation. Health Serv Res. (1988) 23:1. PMC1065486 3130322

[8] Tomita N Yoshimura K Ikegami N. Impact of home and community-based services on hospitalisation and institutionalisation among individuals eligible for long-term care insurance in Japan. BMC Health Serv Res. (2010) 10:1–13. 10.1186/1472-6963-10-345 21176165 PMC3024297

[9] Xu H Weiner M Paul S Thomas J Craig B Rosenman M. Volume of home-and community-based Medicaid waiver services and risk of hospital admissions. J Am Geriat Soc. (2010) 58:109–15. 10.1111/j.1532-5415.2009.02614.x 20002513

[10] Forder J. Long-term care and hospital utilisation by older people: an analysis of substitution rates. Health Econ. (2009) 18:1322–38. 10.1002/hec.1438 19206085

[11] Gaughan J Gravelle H Siciliani L. Testing the bed-blocking hypothesis: does nursing and care home supply reduce delayed hospital discharges? Health Econ. (2015) 24:32-44. 10.1002/hec.3150 25760581 PMC4406135

[12] Hermiz O Comino E Marks G Daffurn K Wilson S Harris M. Randomised controlled trial of home based care of patients with chronic obstructive pulmonary disease. BMJ. (2002) 325:938. 10.1136/bmj.325.7370.938 12399344 PMC130059

[13] Deraas TS Berntsen GR Hasvold T Førde OH. Does long-term care use within primary health care reduce hospital use among older people in Norway? A national five-year population-based observational study. BMC Health Serv Res. (2011) 11:1–11. 10.1186/1472-6963-11-287 22029775 PMC3224781

[14] Sands LP Wang Y McCabe GP Jennings K Eng C Covinsky KE. Rates of acute care admissions for frail older people living with met vs. unmet activity of daily living needs. J Am Geriatr Soc. (2006) 54:339–44. 10.1111/j.1532-5415.2005.00590.x 16460389

[15] Naylor MD Hirschman KB Bowles KH Bixby MB Konick-McMahan J Stephens C. Care coordination for cognitively impaired older adults and their caregivers. Home Health Care Serv Q. (2007) 26:57–78. 10.1300/J027v26n04_05 18032200 PMC2504359

[16] Forster AJ Murff HJ Peterson JF Gandhi TK Bates DW. The incidence and severity of adverse events affecting patients after discharge from the hospital. Ann Intern Med. (2003) 138:161–7. 10.7326/0003-4819-138-3-200302040-00007 12558354

[17] Bragstad LK Kirkevold M Hofoss D Foss C. Factors predicting a successful post-discharge outcome for individuals aged 80 years and over. Int J Integr Care. (2012) 12:e4. 10.5334/ijic.691 22371693 PMC3287325

[18] Lv X Zhang X. The influence of community home-based elderly care on the health of the elderly population. Chin J Populat Sci. (2022) 2022:111–125+128.

[19] Costa-Font J Jimenez-Martin S Vilaplana C. Does long-term care subsidization reduce hospital admissions and utilization? J Health Econo. (2018) 58:43–66. 10.1016/j.jhealeco.2018.01.002 29408154

[20] Feng J Wang Z Yu Y. Does long-term care insurance reduce hospital utilization and medical expenditures? Evidence from China. Soc Sci Med. (2020) 258:113081. 10.1016/j.socscimed.2020.113081 32540515

[21] Chen H Ning J. The impacts of long-term care insurance on health care utilization and expenditure: evidence from China. Health Policy Plan. (2022) 37:717–27. 10.1093/heapol/czac003 35032390

[22] Gruber J McKnight R. Why did employee health insurance contributions rise? J Health Econ. (2003) 22:1085–104. 10.1016/j.jhealeco.2003.06.001 14604562

[23] Lei X Lin W. The New Cooperative Medical Scheme in rural China: does more coverage mean more service and better health? Health Econ. (2009) 18:S25–S46. 10.1002/hec.1501 19551752

[24] Feng J. The effect of social insurance on wages: difference by human capital. J Financial Res. (2014) 7:109–23.

[25] Wang Z Feng J. The substitution effect of long-term care insurance on health expenditure and comparison of different compensation modes. China Econ Q Int. (2021) 21:557–76. 10.13821/j.cnki.ceq.2021.02.09

[26] Guralnik JM LaCroix AZ Abbott RD Berkman LF Satterfield S Evans DA. Maintaining mobility in late life. I Demographic characteristics and chronic conditions. Am J Epidemiol. (1993) 137:845–57. 10.1093/oxfordjournals.aje.a116746 8484376

[27] Pan J Shuai Y Sun T Zhang Y Xue X Zhou C. Disability rate and disability scale of China's elderly population: base on the data of the sixth national population census. J Nat Hist. (2012) 28:3–6. 10.14132/j.2095-7963.2012.04.010

[28] Wang JY Li TR. The age mode of elderly disability in China and the disabled population projection. Populat J. (2020) 5:57–72. 10.16405/j.cnki.1004-129X.2020.05.005

[29] Macintyre S Hunt K Sweeting H. Gender differences in health: are things really as simple as they seem? Soc Sci Med. 42:617–24. 10.1016/0277-9536(95)00335-5 8643986

[30] Nathanson CA. Illness and the feminine role: a theoretical review. Soc Sci Med. (1975) 9:57–62. 10.1016/0037-7856(75)90094-3 1093257

[31] Liu E Zhang Q. Study on gender differences of rural disabled elderly and its influence mechanism: based on the 2014 CLHLS data. Soc Security Stud. (2019) 2:49–58.

[32] Behera DK Dash U. Is health expenditure effective for achieving healthcare goals? Empirical evidence from South-East Asia Region. Asia-Pacific J Reg Sci. (2020) 4:593–618. 10.1007/s41685-020-00158-4

[33] Cv I Behera DK Dash U. Participation of older adults in the intra-household decision-making activities: evidence from the longitudinal aging study in India. J Adult Protect. (2021) 23:325–36. 10.1108/JAP-03-2021-0013

